# α-Synuclein Iron-Responsive-Element RNA and Iron Regulatory Protein Affinity Is Specifically Reduced by Iron in Parkinson’s Disease

**DOI:** 10.3390/biom15020214

**Published:** 2025-02-02

**Authors:** Mateen A. Khan

**Affiliations:** Department of Life Sciences, College of Science & General Studies, Alfaisal University, Riyadh 11533, Saudi Arabia; matkhan@alfaisal.edu

**Keywords:** neurodegenerative diseases, Parkinson’s disease, α-synuclein IRE RNA, IRP1, RNA–protein binding, fluorescence, thermodynamics, circular dichroism

## Abstract

α-Synuclein (α-Syn) is implicated in the pathophysiology of Parkinson’s disease (PD) and plays a significant role in neuronal degeneration. Iron response proteins (IRPs) bind to iron response elements (IREs) found in the 5′-untranslated regions (5′-UTRs) of the messenger RNA that encode the α-Syn gene. This study used multi-spectroscopic approach techniques to investigate the impact of iron on α-Syn IRE RNA binding to IRP1. The formation of a stable complex between α-Syn RNA and IRP1 was suggested by fluorescence quenching results. Fluorescence measurements showed that α-Syn RNA and IRP1 had a strong interaction, with a binding constant (*K*_a_) of 21.0 × 10^6^ M^−1^ and 1:1 binding stoichiometry. About one binding site per IRP1 molecule was suggested by the α-Syn RNA binding. The *K*_a_ for α-Syn RNA•IRP1 with added Fe^2+^ (50 μM) was 6.4 μM^−1^. When Fe^2+^ was added, the *K*_a_ of α-Syn RNA•IRP1 was reduced by 3.3 times. These acquired *K*_a_ values were used to further understand the thermodynamic characteristics of α-Syn RNA•IRP1 interactions. The thermodynamic properties clearly suggested that α-Syn RNA binding to IRP1 was an entropy-favored and enthalpy-driven event, with significant negative ΔH and small positive ΔS. For α-Syn RNA•IRP1, the Gibbs free energy (ΔG) was −43.7 ± 2.7 kJ/mol, but in the presence of Fe^2+^, it was −36.3 ± 2.1 kJ/mol. These thermodynamic calculations indicated that hydrogen bonding as well as van der Waals interactions might help to stabilize the complex formation. Additionally, far-UV CD spectra verified α-Syn RNA•IRP1 complex formation, and α-Syn RNA and Fe^2+^ induce secondary structural alteration of IRP1. According to our findings, iron alters the hydrogen bonding in α-Syn RNA•IRP1 complexes and induces a structural change in IRP1. This suggests that iron selectively affects the thermodynamics of these RNA–protein interactions.

## 1. Introduction

Parkinson’s disease (PD) is a progressive neurodegenerative disease characterized by the accumulation of Lewy bodies, which are intracellular neuronal aggregates. Out of all the diseases that exist worldwide, Parkinson’s disease is the second most common neurodegenerative illness. According to the World Health Organization, around nine million people worldwide suffer with Parkinson’s disease (PD), whose prevalence has doubled in the last 20 years [1]. PD’s etiology is still unknown; however, the primary pathophysiology is the high concentration of α-Synuclein (α-Syn) aggregates in Lewy bodies [2,3,4]. α-Syn protein is a 140-amino-acid residue which is naturally disordered in solution but takes on a helical form when exposed to acidic lipid surfaces [5,6]. α-Syn (SNCA) gene mutations that cause familial PD [7,8] and duplication/triplication of the SNCA that cause early-onset PD in affected families [9] further emphasize α-Syn’s role in disease progression. This is further supported by the fact that one of the main causes of dopaminergic degradation in Parkinson’s disease is the misfolding and subsequent accumulation of α-Syn. Since dementia and dominantly inherited PD with a gene dosage effect develop in people with SNCA gene locus expansion, the rate of fibrillization and neurotoxicity is significantly affected by the α-Syn expression level [10]. As the population ages, effective methods for stopping or reversing α-Syn accumulation and neurotoxicity are desperately required to avert an exponential rise in disease.

Since the description of the hereditary variants of PD, α-Syn has been implicated in the pathophysiology of the disease. According to research on animal models, cell biology, neuropathology, and genetics, α-Syn is a crucial protein in the pathophysiology of PD [11]. This protein has the ability to misfold, oligomerize, and form fibrils, which spread throughout the brain’s neurons and cause neuronal death [12,13]. The various types of accumulated α-Syn may trigger diverse mechanisms that affect physiological function or have negative effects. Nonetheless, the majority of biochemical processes that are triggered by these abnormal α-Syn forms may have similar pathways that lead to comparable synaptic dysfunctions. There are still many important unanswered concerns about its biochemical and biophysical function as well as the best way to target this protein in order to stop or halt the progression of PD.

Aggregation of α-Syn and gradual loss of dopaminergic neurons are the neuropathological hallmarks of Parkinson’s disease. As one of the principal degradation mechanisms, autophagy is essential for preserving efficient turnover of proteins and organelles, preserving cell homeostasis, and averting toxicity and cell death [14]. It has also been demonstrated that α-Syn influences lysosomal and autophagic processes in the mitochondria [15]. According to recent research, autophagy processes may be impacted by additional variables, such as APOE4 expression, which is implicated in various neurodegenerative disorders and may also be connected to PD [16]. Blood cells and other tissues, as well as neurons in the peripheral and central nervous systems, express a little acidic protein α-Syn [17]. Endogenous α-Syn has long been believed to be a naturally unfolded monomer; however, it has been demonstrated to occur mostly as a folded tetramer with little to no amyloid-like accumulation potential [18]. The two forms coexist, although pro-aggregating forms may predominate if the tetramer/monomer ratio is out of balance. α-Syn is composed of three primary areas, each of which is in charge of distinct biological characteristics [19]. The positively charged N-terminus amino acid (residues 1 to 60) is rich in lysine residues and distinguished by amphipathic repeats that frequently adopt a helical shape. This amino acid segment is essential for α-Syn’s ability to bind to membranes [20]. The non-amyloid-β-component (NAC), which is made up of residues 61 to 95 (central region), has been found to be the most prone to aggregation. The negatively charged C-terminus, which is represented by amino acid residues 96–140, is implicated in chaperone-like action and Ca^2+^ binding [21]. The abnormal β-sheet shape that α-syn takes on in Parkinson’s disease attracts more monomers to create amyloid fibrils and oligomers. The inclusions are restricted to either the neuron soma, known as LB, or axons, known as Lewy neurites [22].

Metal ions such as iron (Fe), copper (Cu), zinc (Zn), manganese (Mn), and calcium (Ca) play an indispensable role in brain development and metabolism. An imbalance of metal ions is related to pathogenesis of neurodegenerative diseases. The brain’s high calcium (Ca^2+^) content encourages the formation of worm-like fibrils in PD [23]. Although PD is known to cause disruptions in brain iron homeostasis, it is yet unknown how iron and α-Syn disease are related. PD, Alzheimer’s disease, and other neurodegenerative illnesses are linked to brain iron imbalance [24,25]. It is well recognized that one of the primary causes of neuronal death in PD is iron dysregulation in the brain’s substantia nigra. Different iron complexes build up in brain areas linked to cognitive and motor dysfunction as people age. Labile iron concentrations may rise harmfully and cause oxidative damage and cell death if they surpass the cellular iron storage capability [26]. Conformational transformation of α-Syn from the helical form to β-sheet found in Lewy bodies can be catalyzed in vitro by ferric iron [27]. In those with PD, redox-active iron builds up in Lewy bodies. In the substantia nigra, elevated iron loading of ferritin is indicated by increases in iron and a decrease in ferritin [28]. As observed in post-mortem PD brains, prolonged IRP activity may be the source of reduced ferritin production via lowering ferritin synthesis [29].

The 5′-UTR of α-Syn RNA is organized and functionally significant, and its translation is controlled by an IRE [30]. The relevance of α-Syn in Parkinson’s disease (PD) and the existence of IRE mRNA within the 5′-UTR of the α-Syn gene both demonstrate the role of iron in the pathogenesis of the disease. IRP binds to the IRE when iron levels are low. IRP is bound by iron at high iron concentrations, which releases mRNA for translation [31,32]. Thus, iron homeostasis depends on α-Syn levels, and a malfunction in the IRE-mediated regulation system of α-Syn can lead to overexpression, a malfunction in the regulation of iron storage, and, ultimately, iron-mediated oxidative stress, α-Syn accumulation, dopaminergic neuronal death, and symptoms of PD. An approximately 30 nt stem loop known as the IRE, whose function in iron metabolism has been characterized, is a tiny stem-loop structure within the 5′-UTR that influences mRNA translation. Since the translation initiation complex attaches with the m7G cap structure of messenger RNA, the IRE stem-loop structure is located in the range of fifty nucleotides. Its functionality depends on this distance. The downstream cistron’s output is modulated by IRPs through their binding to the IRE. IRE, found in the α-Syn 5′-UTR mRNA, has been demonstrated to be involved in protein translation [33].

Moreover, iron controls the production of proteins with IRE sequence binding to IRPs [32]. Iron-binding proteins have IRE domains that are similar to the α-Syn mRNA transcript [34]. Consequently, α-Syn expression can be managed by the level of metal ions in neurons. Interactions of IRE RNA lead to structural changes in the IRP domains. These structural conformational alterations of IRE binding to IRP were confirmed using crystallization, X-ray diffraction, and CD analysis [35,36,37]. Conformational alterations in initiation factors binding to IRE RNA and the m7G cap structure were also shown by comparable techniques [38,39]. The structural alterations that α-Syn IRE RNA binding causes in IRP1 are unknown. Entropy and enthalpy promote the stability and strength of the α-Syn IRE RNA·IRP1 complex, which helps to generate overall favorable free energy. This requires an understanding of thermodynamics. In this work, we examine how temperature and iron affect the balance of α-Syn IRE RNA binding to IRP1. Thermodynamic analyses revealed that the presence of iron significantly altered the α-Syn IRE·IRP1 complex’s free energy and enthalpy, indicating modifications in hydrogen bonding and general conformational alterations during complex formation. To further understand the structural changes in IRP1, the α-Syn IRE RNA interaction with IRP1 was examined using far-UV CD analysis. The α-Syn IRE RNA·IRP1 complex’s ellipticity changed significantly in response to iron.

## 2. Materials and Methods

### 2.1. Preparation of RNA and Protein

An IRE RNA oligonucleotide sequence for human α-Syn (5′-ACUGGGAGUGGCCAUUCGACG ACAGUGUGGUGUAAAGGAAUUCAUUAGCC-3′) was procured from Metabion International (AG, Planegg, Germany). In order to prevent degradation, RNA samples were stored frozen at −80 °C. As previously mentioned [40], the RNA was folded by heating for 5 min in RNase-free buffer (20 mM HEPES, 0.1 mM EDTA (pH 7.2), 1 mM MgCl_2_, and 100 mM KCl) at 85 °C (melted and reannealed) and then gradually cooled down to room temperature (30 min) [41]. The RNA concentration was determined by measuring the absorbance at a wavelength of 260 nm, using the standard value (40 µg/mL) of RNA as 1. α-Syn IRE RNA’s purity and lack of degradation were confirmed by the absorbance ratio, which had an A_260/280 nm_ of 1.9. Protein (IRP1) was obtained from OriGene (Rockville, MD, USA). Bio-Rad protein assay reagent was used to determine protein concentration following the Bradford procedure [42]. All RNA studies including RNA preparation were conducted in water treated with diethylpyrocarbonate.

### 2.2. RNA Secondary Structure Predictions

To estimate their most stable folded RNA secondary structure in comparison to the IRE RNA of ferritin (H and L), 50-nucleotide α-Syn IRE RNA 5′-UTR motifs utilized for sequence alignments were selected. The way that RNAs fold into 2° and 3° structures can be correctly predicted by a variety of innovative computing programs [43,44]. For the structural models of RNA folding, we employed the α-Syn IRE RNA oligonucleotide (5′-ACUGGGAGUGGCCAUUCGACGACAGUGUGGUGUAAAGGAAUUCAUUAGCC-3′). The RNAFold Web Server predicts the folding of RNA structure [45,46]. Additional secondary structural models are necessary to determine how the amino acid interactions of proteins with the α-Syn IRE RNA nucleotide affect their respective ability to bind to IRP1.

### 2.3. Fluorescence Spectroscopy Measurement

Fluorescence spectroscopy was described as a means of comprehending the α-Syn IRE and IRP1 interaction using a quartz cuvette that has ten-millimeter pathlength. The slits widths of excitation and emission were set at 5 nm. Excitation was carried out for native protein IRP1 λ_ex_ = 280 nm as a control, and IRP1 fluorescence spectra were recorded in the wavelength λ_em_ range of 300–400 nm. To assess the fluorescence of IRP1 in the presence and absence of α-Syn IRE RNA in a ratio of 1:4, we used free IRP1 and α-Syn IRE RNA/IRP1 complexes. To conduct binding assays of the 50 nM IRP1 protein, increasing quantities of the α-Syn IRE RNA (0.0–200 nM) were added to 20 mM HEPES (pH 7.4) buffer, which contained 1 mM MgCl_2_ and 100 mM KCl. After passing through a 0.22 µm filter, samples were cleared of any suspended material. As an indicator of protein–RNA binding, fluorescence emission quenching of IRP1 was observed upon adding α-Syn IRE RNA. The fluorescence was then monitored for 15 min after IRP1 protein was added to α-Syn IRE RNA.

The experiment was conducted at ambient temperature. To maintain the constant required temperature, samples were incubated for fifteen minutes. For all sample binding studies, a thermocouple device within the cuvette was used to regulate the sample temperature (ΔT ± 0.1 °C). After adding α-Syn IRE RNA, changes in IRP1 fluorescence quenching were computed using

ΔF = (F_0_ − F_f_)/F_0_

where F is the fluorescence quench seen in any sample. The fluorescence intensity of IRP1 protein alone (control) is denoted by F_0_, while the fluorescence quenching after adding α-Syn IRE is denoted by F_f_. Measured fluorescence intensities of the α-Syn IRE/IRP complex were corrected by subtracting the fluorescence of α-Syn IRE RNA alone in buffer. The complex’s binding affinity was determined using the corrected fluorescence. The maximum dilutions were less than 5%, and measured fluorescence intensities were adjusted for inner filter effects as needed. The equilibrium binding affinity (*K*_a_ = 1/*K*_d_) was calculated using the normalized fluorescence quench (F/F_max_) value. The fluorescence change for full IRP1 saturation with α-Syn IRE RNA is denoted by F_max_. F_max_ was calculated by extrapolating to the ordinate the Y-intercept of a 1/F versus 1/[α-Syn IRE RNA] plot [37]. The average value of three separate titration trials was provided for each equilibrium measurement. *K*_d_ values were obtained using KaleidaGraph (version 2.1.3; Abelbeck Software, Reading, PA, USA).

### 2.4. Determination of Binding Site

By using direct protein fluorescence titration assays, α-Syn IRE binding to IRP1 was examined. IRP1 protein fluorescence quenching was achieved at 280/332 nm (excitation/emission) by adding progressively more α-Syn IRE RNA in a 1:4 ratio. All solutions (α-Syn IRE/IRP1 complex) were kept at ambient temperature for 15 min to perform this test. When α-Syn IRE RNA was added, the fluorescence of the IRP1 protein was quenched, and any changes in fluorescence intensity in comparison to a control sample were observed. Untreated IRP1 fluorescence was 100%. At each α-Syn IRE RNA/IRP1 molar ratio (R), the fractional quench (Q) was calculated. Data were fitted to an equation to calculate observed fluorescence (F), [Q = F_0_ − F/m], where m is the maximum quench. Q and α-Syn IRE RNA binding are linearly related: Q = [IRP1•α-Syn IRE RNA]/[IRP1]_T_. As previously mentioned, the normalized fitted data were obtained using Scatchard plots [40] for analysis, n*K*_a_ − *K*_a_Q = Q/[C] = Q/[R − Q][IRP1]_T_, where n represents stoichiometry and C is the free α-Syn IRE RNA concentration; [IRP]_T_ is the total IRP concentration and R represents the α-Syn IRE RNA to IRP1 molar ratio. Additionally, Q[α-Syn IRE RNA] versus Q plot slope and intercept provide n (number of binding sites) and *K*_a_ using the Scatchard equation. Fluorescence data fitting was also used to assess the binding constant between α-Syn IRE RNA and IRP1 interaction, and the results are consistent with the *K*_a_ derived from the slope.

### 2.5. Effect of Fe^2+^ on α-Syn IRE RNA•IRP1 Complex

The aforementioned approach was used to further elucidate the influence of iron on α-Syn IRE binding to IRP1. The same iron concentrations (Fe^2+^, anaerobic condition −O_2_) were added to the α-Syn IRE RNA and IRP1 protein solutions. Before measuring fluorescence, all the samples of free IRP1 and α-Syn IRE RNA/IRP1 complexes were kept for 15 min in 20 mM HEPES buffer, (pH 7.2), 1 mM MgCl_2,_ 100 mM KCl, and 5% glycerol at room temperature. All incubations were anaerobic when Fe^2+^ was employed, as previously mentioned [40]. The α-Syn IRE RNA concentration ranged from 0 to 200 nM, while the IRP1 protein concentration was 50 nM. The nitrogen-purged 0.1M HCl solutions used to dissolve FeSO_4_ and prevent oxidation were diluted to 1mM H^+^ in the case of Fe^2+^, and then further diluted 1:100 into the solutions containing α-Syn IRE RNA or IRP1 protein.

### 2.6. Influence of Temperature on α-Syn IRE RNA∙IRP1 Interaction

To investigate the temperature dependence of the α-Syn IRE interaction with IRP1, both with and without iron, a 0.5 mL sample was kept at experimental temperature for 15 min before use. As previously reported for the fluorescence titration above, this assay was conducted using the same conventional experimental technique at six different temperatures (278, 283, 288, 293, 298, and 303K). In all temperature-dependent binding tests, the sample temperature was kept at ΔT ± 0.1 °C, using a temperature controller.

### 2.7. Thermostability Analysis of α-Syn IRE RNA Binding to IRP1

Temperature-dependent binding affinity tests were performed using fluorescence spectroscopy in accordance with the previously reported standard approach to examine the thermostability of the α-Syn IRE RNA∙IRP1 interaction [47]. The contributions of the interacting forces can be characterized by thermostability data of free energy (ΔG), enthalpy (ΔH), and entropy (ΔS) change between α-Syn IRE RNA and IRP1. Van’t Hoff plots were constructed using temperature-dependent equilibrium constants to assess the thermostability between α-Syn IRE RNA and IRP1 in the presence or absence of iron. Temperature-dependent changes in entropy and enthalpy were observed by Van’t Hoff’s plot. Using Van’t Hoff’s isobaric equation, thermostability values were determined using a temperature dependence binding constant (*K*_a_) as follows:

(1) $$ln\;K_{a\;}=-\frac{∆H}{R\;T\;}+\frac{∆S}{R}$$

where *T* (in Kelvin) is the observed temperature and R (8.31 J mol^−1^ K^−1^) is the gas constant. At 5, 10, 15, 20, 25, and 30 °C, respectively, *K*_d_ (*K*_a_ = 1/*K*_d_) was measured. A plot of ln *K*_a_ vs. 1/T provided values for −ΔH/R and ΔS/R from the intercept and slope, respectively. Changes in energy, entropy, and enthalpy all vary in tandem with each reaction. ΔG values involved in the α-Syn IRE RNA∙IRP1 interaction were obtained following Equation (2).

(2) $$∆G=∆H-T∆S\;\;\mathrm{and}\;∆G=-R\;T\;ln\;K_{a}$$

### 2.8. Circular Dichroism (CD) Measurements

To observe the influence of α-Syn IRE RNA on the secondary structure of IRP1, far-UV (190–260 nm) CD spectra were recorded in 1 mm pathlength cuvettes. CD spectra of protein samples were obtained using a spectropolarimeter (Chirascan Plus, Photophysics Co., Surrey, UK). To maintain a constant temperature of 298 K throughout the experiment, we used a temperature controller with continuous nitrogen circulation and a circulating water bath. The instrument was calibrated with D-10-camphorsulphonic acid, in accordance with the manufacturer’s instructions. CD spectra were acquired at a constant IRP1 concentration (100 nM) with the addition of α-Syn IRE RNA at different concentrations (0–500 nM). CD spectra were obtained at an α-Syn IRE RNA to IRP1 molar ratio of 5:1 at a constant IRP1 concentration of 100 nM with the incubation of each sample for 15 min.

The effects of Fe^2+^ (50 μM) on the structural changes in IRP1 protein with α-Syn IRE RNA binding were seen in additional studies conducted under the identical conditions, as previously mentioned. In order to eliminate any suspended debris, all samples were passed through a filter prior to data collection. Every spectrum was obtained using a reaction time of one second and rate of fifty nanometers per minute with a recording range of 190–260 nm, respectively. Each spectrum has an average of three scans, and noise reduction was conducted prior to final CD spectra collection. The contributions from the reference buffer alone and the α-Syn IRE RNA∙IRP1 complex in buffer, if any, were deducted from the corresponding spectra for each spectrum that was obtained. By removing a buffer–buffer background scan from the first protein spectra, the resulting spectra were verified. The SG filter was applied to all spectra with a bandwidth of 60. Every spectrum data set was shown as θ (deg.cm^2^.dmol^−1^) mean residual ellipticity against wavelength (nm) data. The CDNN program was used to calculate the change in far-UV CD spectra-implied secondary structural alteration (α-helix, β-sheet, β-turn, and random coil) in the IRP1 sample in the presence of varying concentrations of α-Syn IRE RNA. As previously mentioned, the prediction algorithms were also used to determine secondary structures from the amino acid sequence [48]. The percentage of protein α-helical composition was calculated using the following relation:

(3) $$\%\alpha -\mathrm{helix}=\left(\frac{{MRE}_{222nm}-2340}{30,300}\right)\times 100$$

### 2.9. Statistical Analysis

This study was performed in at least three replicates, and all data were reported in mean ± D. The two-tailed Student *t*-test was used to analyze differences. *p* < 0.05 was considered to be statistically significant. All data analyses were performed using KaleidaGraph Software (version 2.1.3, Abelbeck, PA, USA).

## 3. Result

### 3.1. Functional α-Synuclein IRE Is Encoded by 5′-UTR Transcript

Secondary structure and conserved sequences define the IRE [49,50]. RNA’s ability to physically fit into protein molecules depends on its structural shape. For RNA to operate, it must fold correctly into particular structures. The 5′-UTR transcripts of PD’s α-Syn have been shown to possess a functional IRE-containing mRNA sequence [51]. The IRE, a 30 nt stem loop whose function in iron homeostasis has already been characterized, is the most well-characterized 5′-UTR mRNA structure which influences eukaryotic translation. Because the ribosomal pre-initiation complex attaches to this region and initiates protein synthesis, the stem-loop structure of RNA is typically found in the range of fifty nucleotides of the m7G cap structure, which is functionally significant. By attaching to the IRE, IRPs control the downstream cistron’s translation. The untranslated region linked to neurological disorders, including the amyloid precursor protein linked to AD and α-synuclein linked to PD, contains a fully functional IRE, which our lab and others have discovered [37,52].

**Figure 1:**
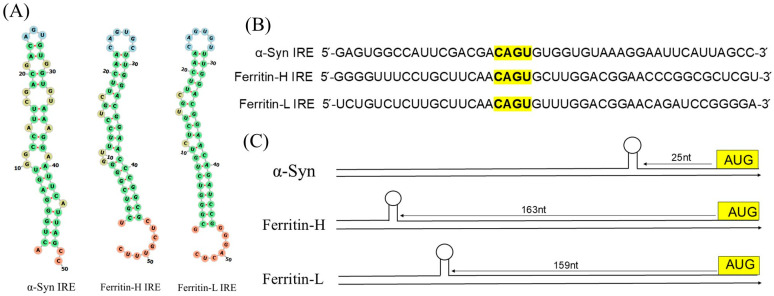
Sequence specificity and RNA structure of IRP1 binding to the IRE motif in the α-synuclein transcript 5′-untranslated region. (**A**) Using the RNAFold software (version 2.1.8), secondary structures of ferritin-H, ferritin-L, and α-Syn IRE RNA were compared. (**B**) Sequences encoding the 5′-UTR-specific IRE stem loops in ferritin-H, ferritin-L, and α-Syn IRE RNA were compared for alignment. The CAGUGN terminal loops are bolded and highlighted in yellow because the sequences encoding the canonical IRE RNA stem loops in the 5′-UTR of the ferritin-H and ferritin-L chains are aligned to the α-Syn IRE. In bold and highlighted yellow letters, the super-conserved homology between the ferritin-H, ferritin-L, and α-Syn IREs subunits is displayed. (**C**) IRE stem-loop maps of the 5′-UTRs encoded by ferritin-H, ferritin-L, and α-Syn transcripts.

Figure 1 shows 5′-UTR maps in mRNA transcripts of ferritin (H and L) and α-Syn, as well as detailed IRE stem-loop structures and sequences. Unlike ferritin (H and L), the general IRE in Figure 1A displays particular IRE stem loops located in the 5′-UTR mRNA transcripts associated with α-Syn. The 5′-UTR IRE structure of α-Syn is similar to IRE structures, which regulates transferrin receptor (TfR) mRNA stability and Fe-dependent ferritin (H and L) translation. The structure and sequence of individual rings of IREs are substantially conserved in mammals. IREs are usually made up of a stem-loop element and C-bulge separated apical loop motif from a lower stem [49,53,54,55]. All IRE RNA has a short (9–10 bp) double-stranded helix with an unpaired C in the center that creates a bulge. Different IRE mRNA sequence variations are relatively minor because all IREs use the same terminal loop and C-bulge sequence to create the RNA A-helix. α-Syn 5′-UTR IRE (50 nt) was used to predict the secondary structure of RNA. To obtain the RNA sequence in dot-bracket format, the CentroidFold technique was utilized. The anticipated secondary structure of α-Syn IRE RNA is displayed in Figure 1A. The RNA structure’s folding was predicted using RNAFold WebServer. α-Syn IRE RNA’s secondary structure is predicted mostly by hairpin loops and base-paired stems. A minimum of −6.40 kcal/mol was free energy (ΔG). Comparable to the 5′-UTR ferritin (H and L) IRE structure is the anticipated secondary structure of α-Syn IRE RNA [41]. The mRNAs of ferritin (H and L) have a canonical IRE, while α-Syn IRE RNA has an atypical 5′-UTR IRE.

Figure 1B displays the alignments of the 5′-UTR transcripts for α-Syn IRE RNA that encode IRE RNA stem loops in comparison with ferritin (H and L) IRE RNA structure. α-Syn IRE RNA 5′-UTR aligns with other 5′-UTRs that have an atypical or canonical IRE. The conserved nucleotides in the CAGUGN motif and an unpaired bulge cytosine were found to be highly similar to the α-Syn 5′-UTR IRE structure. It was hypothesized that, in contrast to the standard IREs, the IRE structure found within α-Syn 5′-UTR RNA would fold into a stem-loop structure. It has been reported that both the IRE structures, atypical and canonical, interact with IRP1 [10,56]. The ferritin 5′-UTR is an example of this homology; it encodes a region that is comparable to the IRE but different from the known α-Syn IRE [57]. A distinct 5′-UTR stem-loop structure of IRE variation, which attaches to IRP1, is encoded by each mRNA. Alignments showed that ferritin (H and L) IRE RNA sequences and α-Syn IRE RNA were more than 70% similar in this 5′-UTR region. The predicted AGU/AGA tri-loops are essential for binding to IRPs and for the suppression of translation, and ferritin 5′-UTR IRE loop-domain CAGUGN is the main point of homology [56,58].

α-Syn 5′-UTR IRE maps of RNA structure in relation to ferritin (H and L) IRE structures are shown in Figure 1C. Identifying the IRE inside the mRNA UTRs is essential. The length of the IRE from the m7G cap mRNA structure and AUG start site may affect how well 5′-UTR is regulated [32]. These distances of IRE from the start codon and from the m7G cap structure vary depending on the type of gene. On the other hand, sequence variants of IRE in other mRNAs from the same species differ considerably more than ferritin mRNA. One instance of a unique α-Syn 5′-UTR IRE is conserved across species [59]. The longer IRE RNA sequences sometimes have extra base pairs in the stem below the C-bulge. Although the IRE structure binds with an nM affinity to IRP and is influenced by Fe at higher quantities, all of the 5′-UTR IREs boost ferritin protein synthesis at high Fe levels, impeding the binding of ribosomes, whereas they inhibit translation at low Fe levels. We identified that the APP 5′-UTR IRE RNA has a functional IRE structure that is comparable to ferritin IRE mRNA [37]. A comparison of the regions of α-Syn 5′-UTR IRE and ferritin 5′-UTR IRE is shown in Figure 1B. According to these alignments, 70% of the sequences between ferritin-H and the APP IRE sequences were similar. To assess the whole activities of this distinct iron-sensitive region in the α-Syn 5′-UTR IRE RNA, many independent transfection experiments were conducted [34].

### 3.2. Iron Weakens α-Syn IRE RNA’s Affinity to IRP1

**Figure 2:**
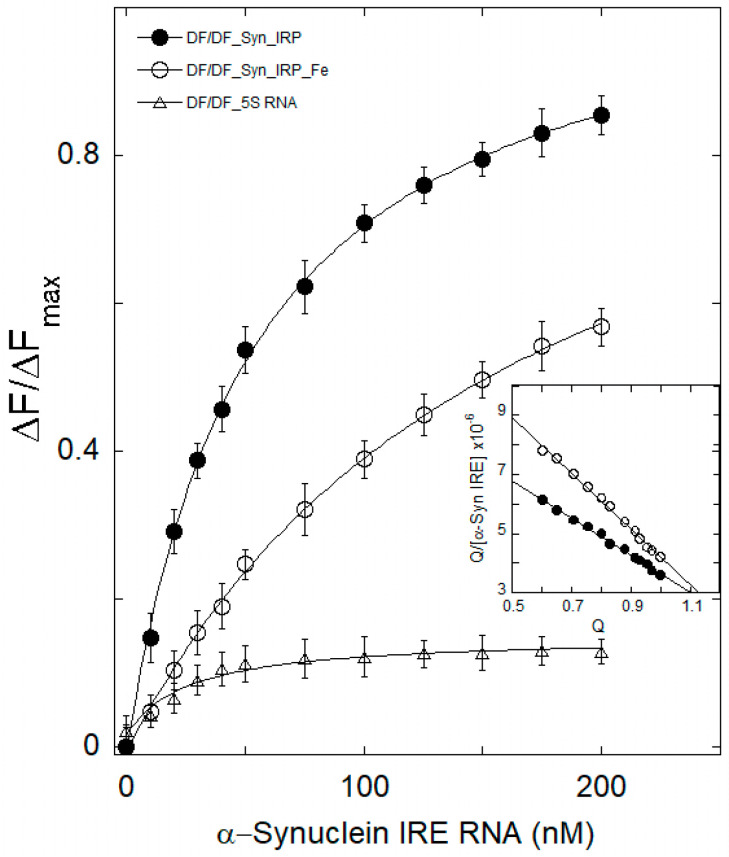
The binding affinity of α-Syn RNA with IRP1 is influenced by iron. Direct fluorescence titration of α-Syn RNA at 25 °C was used to measure the affinity of α-Syn IRE RNA for IRP1 protein. Fluorescence intensity measurements of α-Syn RNA (―●―) and α-Syn RNA-Fe (―○―) binding to IRP1. α-Syn RNA was incubated with IRP1 protein after being melted and annealed before each titration. The excitation maximum for protein titration was 280 nm, whereas the emission maximum was seen at 332 nm. Various amounts of α-Syn RNA were incubated with 50 nM IRP1 to prepare the samples. The stem-loop oligonucleotide (5S RNA), which was employed as a negative control, was not bound by IRP1. A matching Scatchard analysis of the titration data is shown in the inset. The hypothesized curves fit the solid lines. The data are the average of three independent experiments and the average value of the data is reported. Error bars indicate mean ± SD.

To identify the complex formation and determine different binding parameters for the protein–RNA interaction, intrinsic fluorescence intensity can be reported when a complex forms between proteins and RNA. By directly titrating α-Syn IRE RNA with a limited IRP1 concentration, an interaction between α-Syn IRE and IRP1 was ascertained under equilibrium circumstances. Fluorescence emission of the α-Syn IRE∙IRP1 complex in the absence and presence of Fe^2+^ revealed that α-Syn IRE RNA concentration-dependently decreases the fluorescence intensity of IRP1 (Figure 2). The addition of Fe further decreases the fluorescence intensity of the α-Syn IRE∙IRP1 complex. The insertion of α-Syn IRE RNA significantly reduced the fluorescence intensity of IRP1 protein. This difference resulted from structural alterations, including the folding of the IRP1 protein molecule, induced by α-Syn IRE RNA binding. Fluorescence quenching of IRP1 was observed when the α-Syn IRE RNA concentration increased. The peak for the native IRP1 protein was located at 334 nm. On the other hand, IRP1 fluorescence clearly decreased as the quantity of α-Syn IRE RNA increased, indicating the development of an α-Syn IRE RNA•IRP1 complex.

The normalized fluorescence intensity curve of α-Syn IRE∙IRP1 and α-Syn IRE ∙IRP1-Fe^2+^ is displayed in Figure 2. The quantity of bound α-Syn IRE to IRP1 suggests the development of a complex that was thought to be related to the amount of protein fluorescence quenching. Tryptophan, tyrosine, and phenylalanine were among the fluorescence groups that were encapsulated, which resulted in fluorescence quenching. When α-Syn IRE RNA binds to IRP1, the protein structure may be packed more tightly, or the aromatic amino acids may be brought closer together. This may cause a spectrum shift in fluorescence emission by altering the electronic distribution in the fluorophores [60]. One of the main tools for the quantitative study of fluorescence quenching is the Scatchard equation. It gives the correlation between the quencher concentration and the protein fluorescence change with and without a quencher. The binding constant and stoichiometry were calculated using the Scatchard equation. IRP1 has a 1:1 stoichiometric titration for binding α-Syn IRE RNA, which was used to determine each IRP1 preparation fractional activity for stoichiometric titration. The RNA concentration was 15 times more than the apparent *K*_d_, and the ratio of IRE RNA:IRP1 fluctuated in range of about 4-fold.

The Scatchard plot of α-Syn IRE RNA’s interaction with IRP1 is displayed in the inset of Figure 2. The binding affinity (*K*_a_) and stoichiometry (n) of α-Syn IRE∙IRP1 binding were determined from the slope and intercept, respectively, of Q versus Q/[α-Syn IRE] × 10^−6^. When α-Syn IRE RNA interacted with IRP1, the binding affinity was 20.8 × 10^6^ M^−1^. While the n value for α-Syn IRE RNA/IRP1-Fe^2+^ was about 1.2, indicating slightly more than one binding site per IRP1, the n value for α-Syn IRE RNA/IRP1 was found to be 1.1, suggesting that there is approximately one binding site per IRP1 molecule for α-Syn IRE RNA.

In contrast to α-Syn IRE RNA/IRP1, the *K*_a_ value for α-Syn IRE RNA/IRP1-Fe^2+^ (6.4 × 10^6^ M^−1^) was lower, indicating a decreased binding affinity of α-Syn IRE RNA to IRP1 when iron is present. This can be the result of a less advantageous chemical interaction or less accessible binding sites on IRP1. These variations in *K*_a_ and n values between α-Syn IRE RNA and IRP1 demonstrate how Fe^2+^ affects how α-Syn IRE RNA binds to IRP1. Non-linear analysis of the fluorescence data provided values for binding affinity (*K*_d_ = 47.6 ± 1.7 nM) in good agreement with the *K*_d_ value for α-Syn IRE∙IRP1 binding, as obtained using Scatchard plots. The equilibrium constants for α-Syn IRE∙IRP1 interactions were reported in *K*_d_ (*K*_a_ = 1/*K*_d_).

In order to better examine how iron affects the stem-loop structure of α-Syn IRE RNA with regard to its binding affinity with IRP1, both were incubated anaerobically (−O_2_) with 50 μM of Fe^2+^ added. Concentration-dependent decreases in the fluorescence intensity of native IRP1 were observed at emission λ_em_ = 332 nm (λ_ex_ = 280 nm) with the addition of a-Syn RNA in the presence of Fe^2+^. The fluorescence intensity curves of α-Syn IRE∙IRP1 and α-Syn IRE∙IRP1-Fe^2+^ were substantially different, as seen in Figure 2. When iron was added to the α-Syn IRE RNA•IRP1 complex, the normalized fluorescence curve was considerably reduced. This discrepancy developed because the α-Syn IRE RNA•IRP1 complex became unstable due to structural alterations brought on by iron binding. When iron was added, α-Syn IRE RNA binding to IRP1 was decreased approximately 3.3 times at 25 °C (α-Syn IRE RNA•IRP1-Fe^2+^, *K*_d_ = 157 ± 7.7 nM; α-Syn IRE RNA•IRP1, *K*_d_ = 47.6 ± 1.7 nM) (Table 1). However, under the same experimental conditions, IRP1 did not bind 5S RNA (a negative control), a 30 nt stem-loop structure. This suggests that α-Syn IRE RNA specifically recognizes IRP1 binding pockets, as observed previously for ferritin IRE and APP IRE RNA [37,40]. These findings offer quantifiable evidence in favor of the stem-loop α-Syn IRE RNA structure’s particular binding affinity for IRP1. APP and ferritin IRE RNA/IRP1 complexes are among the other IRE RNAs that have been shown to be destabilized by iron [40].

### 3.3. Therma Stability Characterization of α-Syn IRE RNA•IRP1

**Figure 3:**
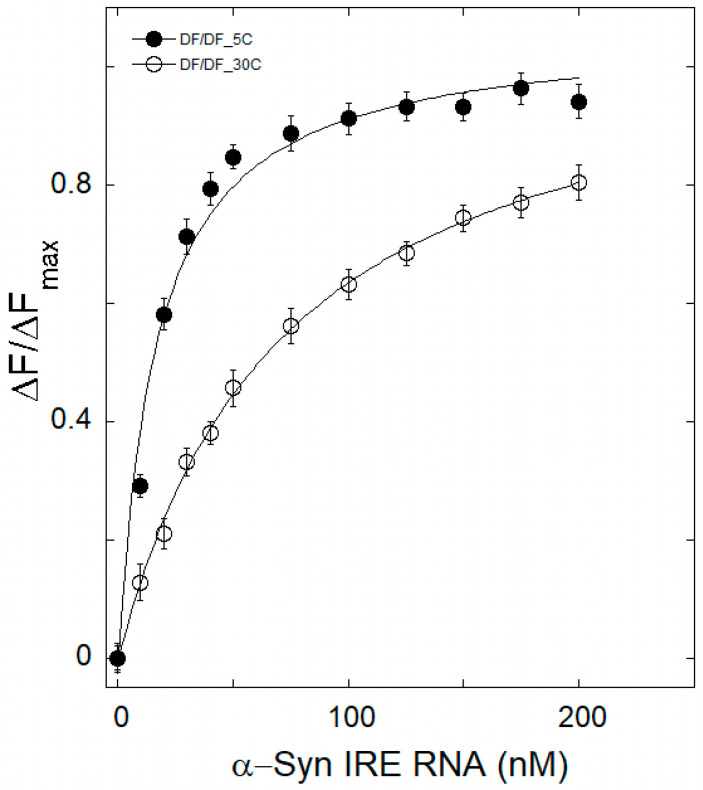
A representative plot of the temperature-dependent binding of α-Syn IRE RNA to IRP1. α-Syn RNA was incubated with IRP1 protein after being melted and annealed before each titration. IRP1 protein (50 nM) was treated with different amounts of α-Syn RNA at 5 °C (―●―) and 30 °C (―○―). The wavelengths for excitation and emission were 280 and 332 nm, respectively. Curves are fitted to the solid lines. Three separate experiments were averaged to produce the data, and the average value of the data is provided. Mean ± SD is displayed in the data.

**Figure 4:**
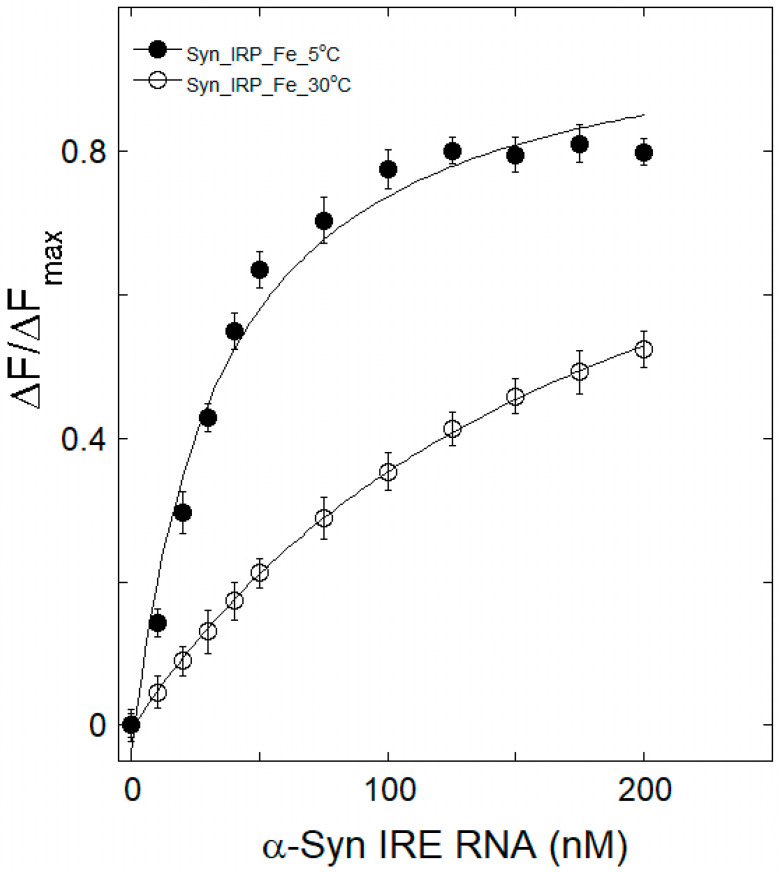
A representative plot of the temperature-dependent binding of α-Syn IRE RNA to IRP1 when iron is present. Before every titration, α-Syn RNA was melted, annealed, and incubated with IRP1 protein. The 50 nM IRP1 protein was treated with different α-Syn RNA concentrations and 50 μM Fe^2+^ at 5 °C (―●―) and 30 °C (―○―). The wavelengths for excitation and emission were 280 and 332 nm, respectively. Curves are fitted to the solid lines. Three separate trials were used to calculate the average value of the data, which is then published. The displayed data are the mean ± SD.

The mechanism of quenching in an α-Syn IRE RNA•IRP1 interaction can be inferred from its temperature dependence. Samples of α-Syn IRE RNA•IRP1 were heated to temperatures ranging from 5 to 30 °C in order to provide insight into how thermal treatment affects the structure of the protein and RNA complex. For this reason, several temperatures were considered in research involving the quenching of fluorescence. The temperature-dependent fluorescence intensity measurement curves of α-Syn IRE∙IRP1 binding at 5 °C and 30 °C, both with and without iron (50 μM), were evaluated by analyzing the data in Figure 3 and Figure 4 and are summarized (*K*_d_) at various temperatures in Table 1. It is evident that a higher *K*_d_ value was seen as the temperature rose, and this kind of temperature-dependent *K*_d_ change suggests that α-Syn IRE RNA and IRP1 formed a dynamic complex. *K*_d_ rises as temperature rises, suggesting that the α-Syn IRE RNA•IRP1 interface has a dynamic mode of interaction. The results of fluorescence investigations conducted at various temperatures showed that, in the absence of iron, the *K*_d_ rose with temperature, rising from 5 °C (*K*_d_ = 13.5 ± 0.6 nM) to 30 °C (*K*_d_ = 63.8 ± 3.2 nM). The α-Syn IRE RNA/IRP1 complex is stabilized at lower temperatures.

**Figure 5:**
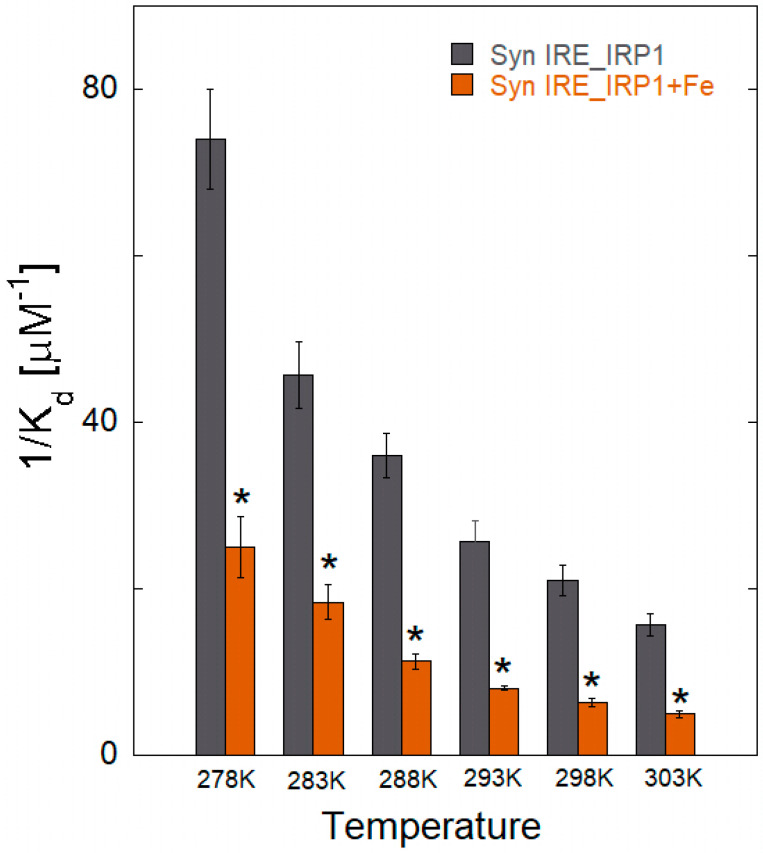
Comparing the binding affinities of IRP1 and α-Syn RNA at different temperatures with and without 50 μM Fe^2+^. Three separate time averages are used as the data, and the standard deviation is used to calculate the error as mean ± SD. Differences were calculated using a two-tailed Student *t*-test, with significant differences from Syn IRE/IRP1 with added Fe^2+^ (*, *p* < 0.05) at different temperatures.

The *K*_d_ value of the α-Syn IRE RNA∙IRP1 binding at 30 °C was greater than that at 5 °C, according to fluorescence quenching data analysis (Table 1). The *K*_d_ value of the α-Syn IRE RNA∙IRP1-Fe^2+^ complex rose from 29.6 ± 1.5 nM to 198.7 ± 7.4 nM when temperature increased (5 °C to 30 °C). Within the temperature range under investigation, the *K*_d_ values of α-Syn IRE RNA/IRP1 rose with the addition of Fe^2+^ (50 μM). According to the binding data, α-Syn IRE∙IRP1 binding with addition of iron had a consistently decreased binding affinity compared to α-Syn IRE RNA/IRP1 at all five temperatures (Figure 5). Since it provides information regarding α-Syn IRE RNA•IRP1’s effective mode of interaction, the temperature-dependent binding constant was considered. The fact that the *K*_d_ rises with temperature was clearly visible, suggesting a dynamic manner of interaction for this activity. At all temperatures considered, binding stoichiometry (n) was close to one.

### 3.4. Thermodynamic Characteristics of α-Syn IRE RNA Binding to IRP1

**Figure 6:**
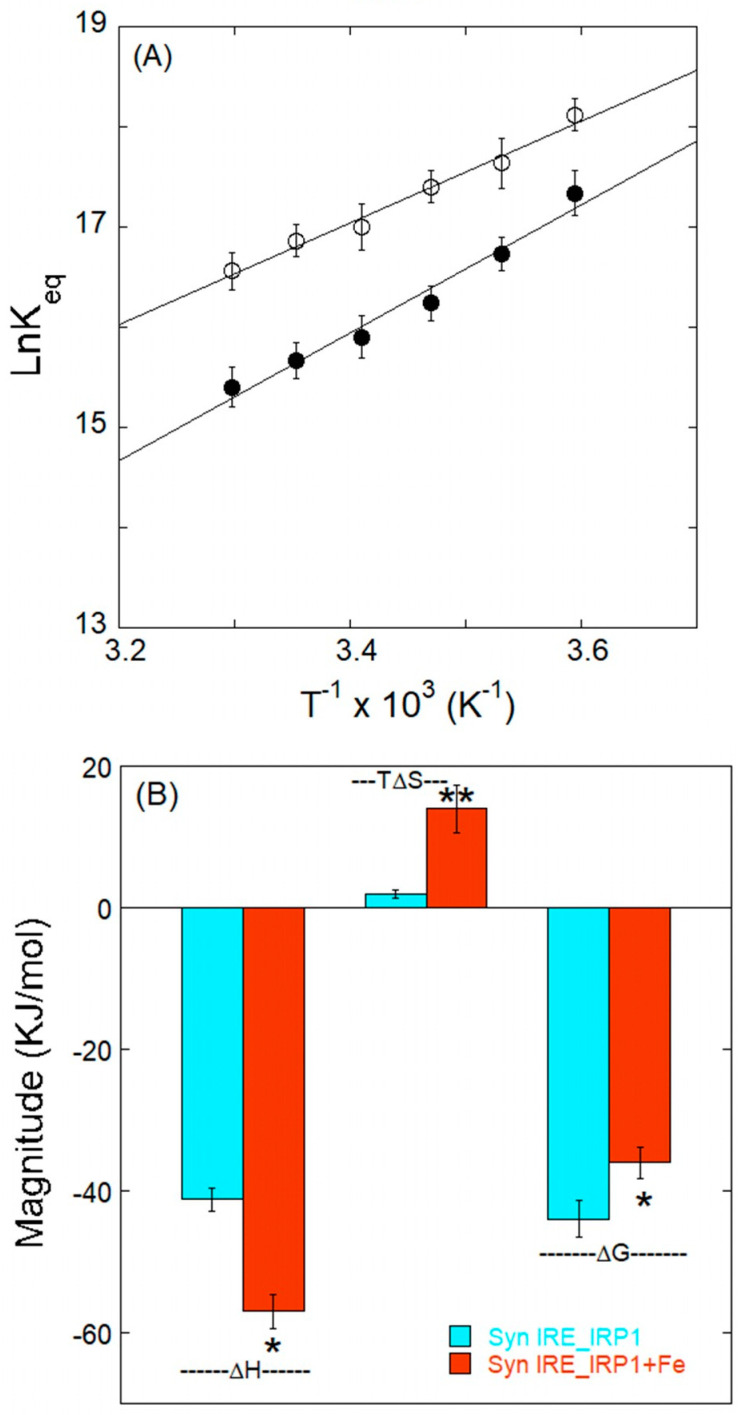
Iron-induced thermodynamics of α-Syn RNA binding to IRP1. (**A**) The stability of the α-Syn RNA/IRP1 (―●―) and α-Syn RNA/IRP1-Fe^2+^ (―○―) complex is influenced by iron. The slope and intercept of the temperature-dependent Van’t Hoff plot were used to compute the thermodynamic parameters (ΔH and ΔS). The thermodynamic characteristics of the binding of α-Syn RNA to IRP1 are shown in (**B**). In the bar plot, ΔG, ΔH, and entropy’s contributions to the free energy (−TΔS) of the binding of α-Syn RNA to IRP1 are shown. The average value of the data is presented, and the data are the average of three separate tests. The shown data are mean ± SD. Differences were calculated using 2-tailed Student’s *t*-test (* *p* < 0.05; ** *p* < 0.01).

The thermodynamic properties of static complex formation were investigated to further understand the mechanism involved in the α-Syn IRE RNA•IRP1 interaction. The α-Syn IRE∙IRP1 complex’s stability was assessed using thermodynamic parameters. We explored the thermodynamics of α-Syn IRE∙IRP1 binding by estimating *K*_d_ at different temperatures, which helped us determine the nature of the forces responsible for complex formation in the absence and presence of Fe^2+^. Linear analysis of Van’t Hoff plots was performed using a graph with 1/T on the x-axis and ln*K*_eq_ values on the y-axis (Figure 6A).

Linear analysis of the Van’t Hoff plot provided values for ΔG, ΔH, and ΔS, which makes use of the experimental temperature (T), the gas constant (R), and the computed *K*_a_ values in Table 1. The Van’t Hoff plot’s slope yields ΔH, while its intercept yields ΔS. Table 2 and Figure 6B show ΔG, ΔH, and TΔS values that were subsequently estimated from the equation. The thermodynamic data analysis showed that the interactions are enthalpically driven and favored by conformational entropy, with a positive ΔS (7.0 ± 0.4 J/mol/K) and a high negative ΔH (−42.2 ± 2.3 kJ/mol) [47]. The thermodynamic properties of α-Syn IRE∙IRP1 and α-Syn IRE∙IRP1-Fe^2+^ are compared in Table 2. The ΔH and ΔS of binding for the α-Syn IRE RNA/IRP1 are considerably altered by the addition of iron, reaching −53.0 ± 4.6 kJ/mol and 48.0 ± 2.7 kJ/mol, respectively. Additionally, negative ΔH (<0) and positive TΔS (>0) demonstrated that the involvement of hydrogen bonds as well as van der Waals interactions may be important in stabilizing the α-Syn IRE RNA∙IRP1 complex. According to previous research, the dominant noncovalent forces in protein binding to ligands can be linked to the magnitude and sign of the individual or combined values of enthalpy and entropy. According to the thermodynamic values shown in Table 2, α-Syn IRE RNA and IRP1 spontaneously connect through dominant hydrogen bonding and van der Waals forces.

The thermodynamic measure of the spontaneity of a binding reaction is the Gibbs free energy change (ΔG). A negative ΔG value suggests that, in normal circumstances, α-Syn IRE∙IRP1 complex formation occurs spontaneously. The thermodynamic parameters of α-Syn IRE RNA binding to IRP1 with or without iron are displayed in Figure 6B. The variables from Table 2 and Equation 2 were used to estimate value of ΔG at 25 °C. The computed value of ΔG was −43.7 ± 2.7 kJ/mol and −36.3 ± 2.1 kJ/mol for the α-Syn IRE∙IRP1 and α-Syn IRE∙IRP1-Fe^2+^. The greater spontaneous binding affinity of α-Syn IRE RNA’s interaction with IRP1 in relation with iron is indicated by the more negative ΔG for α-Syn IRE RNA∙IRP1 as opposed to α-Syn IRE RNA∙IRP1-Fe^2+^. This might be because of a more advantageous molecular connection, which is less noticeable when iron is present. The structural and environmental differences between α-Syn IRE RNA/IRP1 and α-Syn IRE RNA/IRP1-Fe^2+^ can account for the variation in ΔG values between these two samples. In contrast to the absence of iron, Fe^2+^ may provide a distinct microenvironment or steric barrier that influences the interaction between α-Syn IRE RNA and IRP1, leading to a less negative ΔG. Because they shed light on the binding process’ energetics and aid in comprehending the molecular interactions underlying the fluorescence quenching mechanism, these findings are noteworthy. For applications where it is important to determine how well medicinal drugs connect to their targets, the spontaneity of the binding process is essential.

### 3.5. Conformational Alteration of IRP1 upon Binding to α-Syn IRE RNA

**Figure 7:**
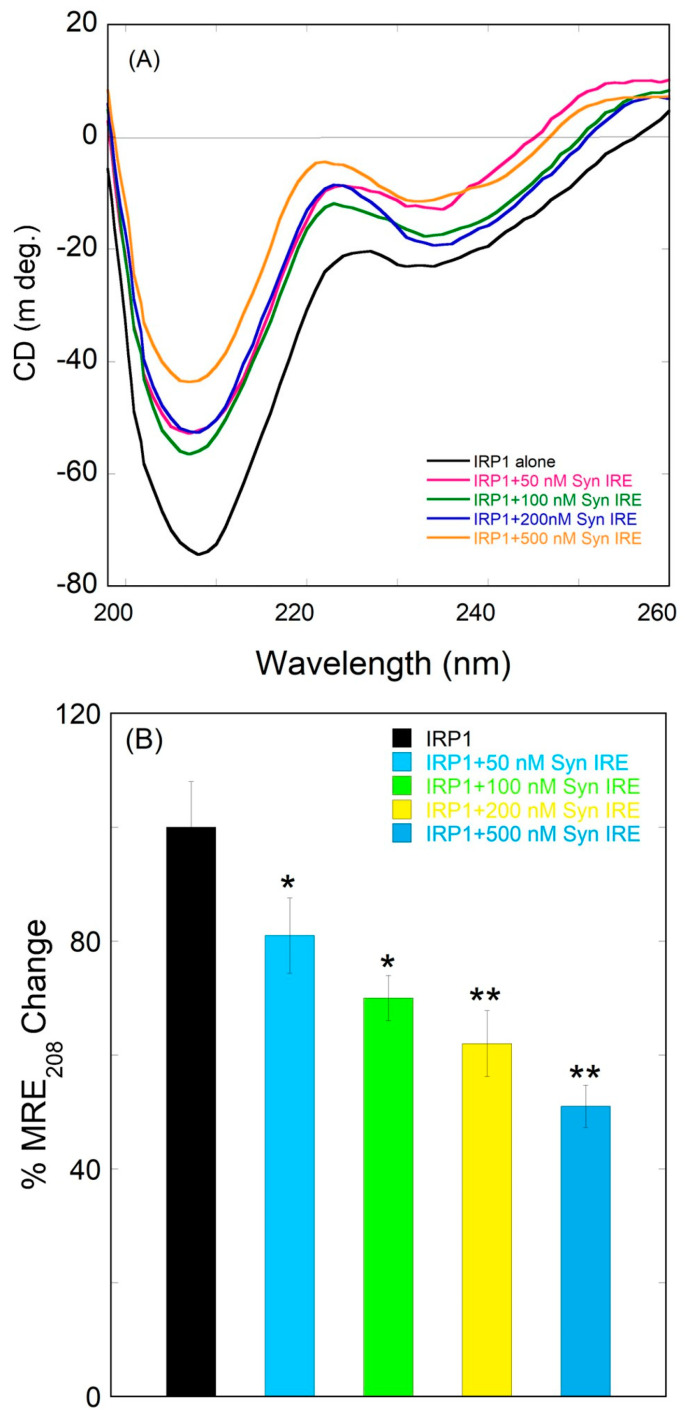
Far-UV CD spectra of the IRP1 protein after adding different amounts of α-Syn RNA. The IRP1 protein’s ellipticity is considerably altered by the addition of α-Syn RNA. (**A**) The far-UV CD spectra for IRP1 (100 nM) with different amounts of α-Syn RNA (0–500 nM). Panel (**B**) displays a bar plot that compares the CD spectral minimal MRE_208nm_ value. The data are the average outcome of three separate experiments. A two-tailed Student *t*-test was used to calculate differences (* *p* < 0.05; ** *p* < 0.01). Error bars indicate SD.

Far-UV CD provides crucial information regarding protein conformation features at the secondary structural level. The effects of α-Syn IRE on the conformation and secondary structure of IRP1 were investigated. Protein secondary structural changes are reflected in changes in the protein’s far UV-CD spectra. In this work, we first looked at the CD spectrum of the native IRP1 protein by itself. We investigated the structural changes that IRP1 underwent when it bound to α-Syn IRE RNA. Figure 7A shows the far-UV CD spectra of free IRP1 (100 nM) and the α-Syn IRE∙IRP1 complex with varying α-Syn IRE RNA (0–500 nM) concentrations. The presence of an α-helical conformation is indicated by the far-UV CD of free IRP1, which shows strong minima at 208 nm and exhibits a modest peak at 218 nm that is indicative of the protein’s β-sheet conformation. The natural IRP1 CD spectra are shown in Figure 7A, exhibiting a peak at about 208 nm that is typical of an α-helix. Therefore, it is evident from this peak at about 208 nm that IRP1 is a protein rich in α-helices. Protein conformation alterations at the secondary structure level (α-helix, α-sheet, α-turn, and random coil) have been examined with the help of the far-UV CD measurements [61,62].

The CD spectra of IRP1•α-Syn IRE RNA and free IRP1 are shown in Figure 7, which indicates that α-Syn IRE RNA causes secondary alterations in IRP1; a change in band intensity is evident, but there is no discernible peak shift. This upward movement of the CD spectra of IRP1 caused by α-Syn IRE RNA indicates that binding of α-Syn IRE induces secondary structural alteration. It is evident from the CD analysis that free IRP1 and bound IRP1∙α-Syn IRE RNA differ structurally. The insertion of α-Syn IRE RNA significantly affects IRP1’s structure. After binding to the α-Syn IRE RNA, the ellipticity value of IRP1 changes in the far-UV CD spectra, indicating a reduction in α-helices and enhancements in the β-sheet conformation. The band intensity without a discernible shift in IRP1 peak location suggests that secondary structural alterations are induced by α-Syn IRE RNA binding. When α-Syn IRE RNA is added, IRP1’s ellipticity increases, which suggests that the secondary structure is being lost. The percent MRE_208_ changes in free IRP1 and the α-Syn IRE∙IRP1 complex are displayed in Figure 7B.

**Figure 8:**
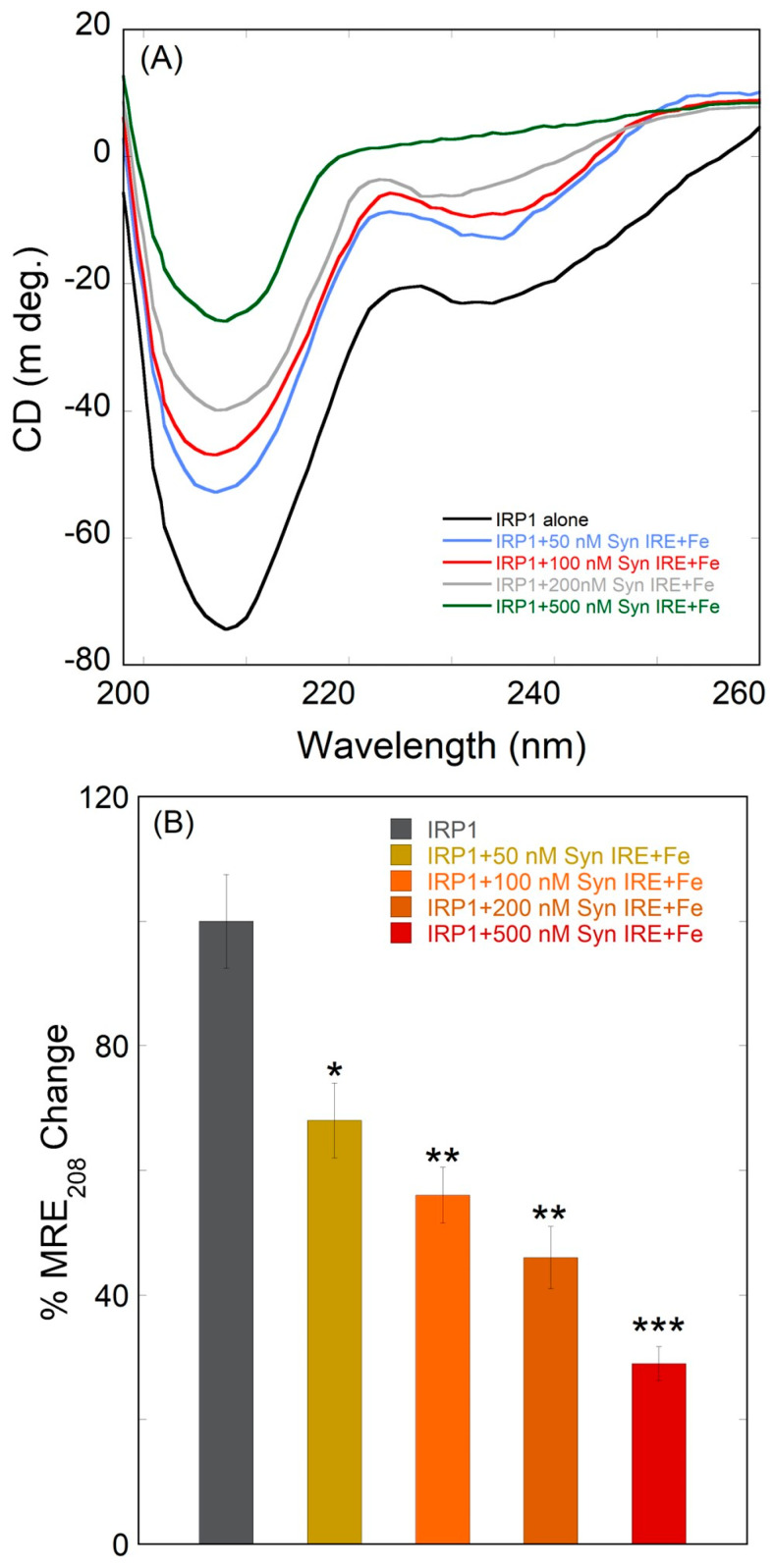
The CD spectra of IRP1 binding to α-Syn RNA are influenced by iron. The ellipticity of the α-Syn RNA/IRP1 complex is considerably altered by the addition of iron (50 μM). (**A**) The far-UV CD spectra for IRP1 (100 nM) with α-Syn RNA (0–500 nM) in the presence of iron. (**B**) Bar plot of α-Syn RNA/IRP1-Fe^2+^ versus CD spectrum minimal MRE_208nm_ value. The data are the mean result of three different experiments. Differences were calculated using a two-tailed Student *t*-test (* *p* < 0.05; ** *p* < 0.01; *** *p* < 0.001). SD is indicated by error bars.

Far-UV CD spectra were used to further examine the impact of iron on the secondary structure of IRP1 binding to α-Syn IRE RNA. Circular dichroism and fluorescence spectroscopy, as illustrated in Figure 8A, are in agreement with the binding results; the intensity of IRP1 further dropped with the addition of Fe^2+^. Characteristics of an alpha helix were visible on one significant negative peak at around 208 nm curves of the IRP1∙α-Syn IRE RNA CD spectra treated with Fe^2+^. The band intensity of IRP1 steadily declined as the concentration of α-Syn IRE RNA increased in the presence of iron. In line with this, the IRP1 β-sheet content rose while the α-helix content steadily declined.

Bar plots demonstrating the effect of Fe^2+^ on the ellipticity changes at 208 nm of IRP1’s interaction with α-Syn IRE are displayed in Figure 8B. These findings demonstrated that, in the presence of Fe^2+^, IRP1 displayed more structural modification with α-Syn IRE RNA. According to these findings, the IRP1•α-Syn IRE RNA complex’s secondary structure composition changed significantly more when Fe^2+^ was added. As previously mentioned, the secondary structural contents were estimated [48,63,64]. Following α-Syn IRE RNA treatment at 500 nM, IRP1’s secondary structure contents directly dropped by 50% of its α-helix content. When α-syn IRE RNA was added, we saw a drop in the α-helicity values of IRP1. These modifications to the secondary structure show how the insertion of α-Syn IRE RNA causes specific structural alterations in IRP1. In contrast, the addition of 500 nM α-Syn IRE RNA and 50 μM Fe^2+^ caused a direct 70% drop in IRP1 structural contents. α-Syn IRE RNA binding to IRP1 underwent a significant structural change due to iron. When iron was added, we saw a loss of α-helicity in the α-Syn IRE RNA/IRP1 complex. These secondary structure alterations show that the complex changes structurally as a result of the iron addition. Given this significant structural alteration, it is possible that this conformational shift has a regulatory function and that α-Syn IRE RNA interacts with IRP1 through an α-helix.

## 4. Discussion

α-Syn IRE RNA in its 5′-UTR binding to IRP1 was examined through biophysical experiments. It has been shown that IREs of ferritin and APP interact significantly with IRP1, and that iron destabilizes this complex [37,40]. In order to control α-Syn translation regulated by IRE in the 5′-UTR, accounting for the iron-dependent translational control, we extended the restrictions of similar IREs in the 5′-UTR in its encoding mRNA by using prior information of ferritin and APP RNA binding to IRP1. When compared to when iron was not added, α-Syn IRE RNA’s affinity to IRP1 was reduced about 3.3 times. The affinity of iron, on the other hand, was reduced by about 17 times for ferritin and 3.5 times for APP RNA for IRP1 [37,40]. The equilibrium stability of α-Syn IRE RNA is approximately 14 times greater than that of ferritin IRE RNA when iron is present. While the binding affinities of APP IRE RNA and α-Syn IRE RNA to IRP1 are similar, ferritin IRE RNA has a far tighter binding constant, even when iron is absent, than either of these RNAs.

Both complexes contain identical IRPs, and the only difference is that the 5′-UTR IRE structures of ferritin and α-Syn are reflected in the difference in stability of the IRE∙IRP1 seen here. The differences in the binding of IRP1 and IRP2 to IREs on the 5′-UTR and 3′-UTR in vitro and the several-fold differential mRNA expression are then at least partially explained by IRE RNA structural variations [41]. Given that ancient ferritin IREs in the 5′-UTR and α-Syn 5′-UTR IRE RNA evolved more recently [30,65], the smaller iron response and reduced α-Syn IRE RNA∙IRP1 stability may be a result of the IRE RNA’s shorter evolutionary fine-tuning time. Nonetheless, the physiological role of each encoded protein may also be reflected in the distinct iron reactions of the two IRE RNAs. For instance, significant variations in α-synuclein synthesis may be harmful to oxidative metabolism in cells, which could account for the comparatively few synthesis modifications brought on by iron. On the other hand, ferritin mRNA translation enables cells to react to significant changes in iron through significant changes in ferritin production [66].

α-Syn IRE RNA and IRP1 formed a complex, as demonstrated by fluorescence experiments. Fe^2+^ destabilizes complexes of α-Syn IRE RNA and IRP1. α-Syn IRE•IRP1’s stability is affected by Fe^2+^, which suggests that the complex can detect changes in labile Fe^2+^ concentrations that lead to weakened RNA•IRP1 connections. IRP1 mediates the increase in 5′-UTR IRE-dependent translation in response to changes in cellular iron [67]. The possible binding forces between different proteins and ligands have been found in numerous studies using thermodynamic characteristics [68]. Our earlier findings of the 5′-UTR IRE structure of APP and ferritin binding to IRP1 have provided insight into the mechanism of their interaction [47].

Interestingly, adding iron caused a considerable change in the ΔG value of the α-Syn IRE RNA•IRP1 complex. α-Syn IRE RNA’s interaction with IRP1 is thermodynamically favored by negative ΔG values. The interaction between α-Syn IRE RNA and IRP1 is beneficial both entropically (4.8%) and enthalpically (96.5%), with a 92% greater enthalpic contribution to ΔG at 25 °C. When iron was added, α-Syn IRE RNA binding to IRP1 had an entropic contribution of 39.4% and an enthalpic contribution of 146%. When iron is present, the enthalpic contribution of α-Syn IRE RNA•IRP1 to ΔG is higher than that of α-Syn IRE RNA associated with IRP1 alone. The thermodynamic parameters’ sign and magnitude influenced the involvement of the protein–RNA bonding forces [68,69]. Furthermore, a substantial negative ΔH indicates that the dominating forces responsible for α-Syn IRE RNA•IRP1 complex formation are hydrogen bonding and van der Waals interaction [68,70]. However, α-Syn IRE RNA•IRP1 complexes with and without iron showed a high TΔS value, indicating the presence of hydrophobic contacts. Thus, these findings further suggest that the α-Syn IRE RNA•IRP1 complex’s stability involves the potential binding forces of hydrogen bonds as well as van der Waals-driven interactions.

ΔG results for the involvement of hydrogen bonding in α-Syn IRE RNA•IRP1 further corroborated our enthalpic results. Fe^2+^ reduced the binding affinity by 3.3 times and the binding free energy to roughly −7.4 kJ/mol for α-Syn IRE RNA binding to IRP1. The ΔG of roughly 5–6 kJ/mol, which is the standard of a hydrogen bond produced between various RNA–protein interactions, is represented by this difference [71]. Iron may cause conformational changes in α-Syn IRE RNA•IRP1 interactions through altering the amount of hydrogen bonding, which destabilizes the complex, according to the ΔG of complex formation. These findings suggest that iron causes a conformational shift in the α-Syn IRE RNA/IRP1 complex, which results in reduced hydrophobic interactions and hydrogen bonding, perhaps reducing binding selectivity.

The α-Syn IRE RNA·IRP1 complex’s selective conformational alterations brought on by iron highlight how sensitive the RNA–protein structure–function link is to its surroundings. Similarly to ferritin and APP IRE RNA binding to IRP1, the α-Syn IRE RNA·IRP1 complex is destabilized by Fe^2+^ due to structural alterations in IRP1 following the α-Syn IRE RNA interaction [37,39]. These α-Syn IRE·IRP1 conformational changes can also be thermodynamically regulated, as has been documented for other RNA–protein interactions [72]. In addition to enabling ribosomal–initiation complex interaction and enhanced expression of α-Syn 5′-UTR IRE mRNA transcripts, the conformational shift facilitates IRP1’s dissociation from the α-Syn IRE RNA·IRP1 complex. The binding of ligands leads to structural changes in proteins, one of the most important methods for examining conformational changes in proteins [73]. To assess random coils, beta sheets, and alpha helices, CD spectra studies were conducted. The findings demonstrated that IRP1 is an α-helix-rich protein, as seen by its distinctive peaks at 208 nm. IRP1’s UV CD spectrum shifts when α-Syn IRE RNA is added, indicating that RNA causes structural changes in IRP1. It is obvious that iron plays a significant role in the α-helix loss of the IRP1 structure because it also causes structural alterations in the α-Syn IRE RNA-IRP1 complex of the CD spectra. The secondary structure could affect the stiff and flexible structures of proteins through the creation of hydrogen bonds [74].

According to CD analysis results, most of the IRP1 could still retain structural integrity at 500 nM of α-Syn IRE RNA and 50 μM of Fe^2+^ or less, but the secondary protein structures were drastically altered. The thermodynamic results indicated that the complex’s structure was altered by variations in the forces involved in the interaction between α-Syn IRE RNA and IRP1, as evidenced by changes in free energy and enthalpy with the addition of iron. Although IRP1 underwent secondary structural modifications, it essentially stayed in its folded shape, which is in line with the findings of thermodynamic and CD studies. All of these results support the idea that iron caused structural alterations in the α-Syn IRE RNA/IRP1 complex, which were probably caused by variations in the quantity of salt bridges and hydrogen bonds.

**Figure 9:**
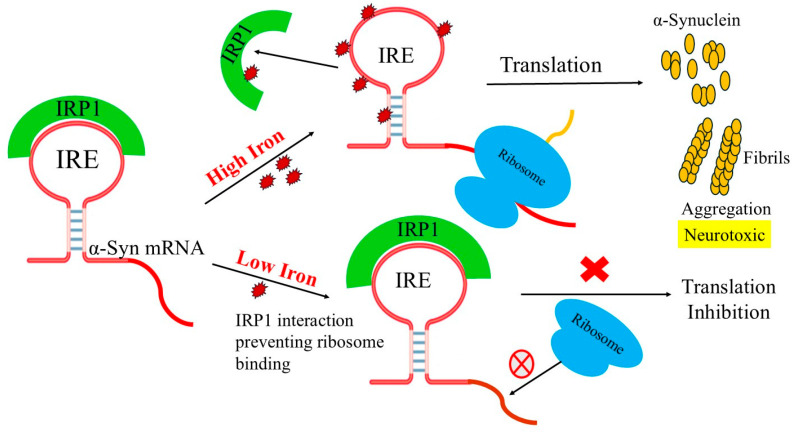
A proposed model for the translational regulation of the α-Syn IRE RNA that is dependent on iron. IRP1 binds to the 5′-UTR of α-Syn IRE RNA more readily when cellular iron levels are low. This binding prevents ribosome contact, which prevents the translation of α-synuclein. IRP1 separates from the α-Syn RNA stem-loop structure at high cellular iron levels, enabling ribosome and initiation factors to bind and enhancing α-Syn RNA translation.

Figure 9 illustrates how Fe^2+^ metabolites affect α-Syn IRE mRNA/IRP1-regulated protein synthesis in a model of α-Syn IRE mRNA riboregulatory function. Iron-regulated neurotoxic α-synuclein protein production is modeled to reflect the physiological iron signal in α-Syn mRNA translation. Adequately raising cellular iron levels reduces α-Syn IRE RNA/IRP1 association, ribosomal assembly, and initiation factor binding, while promoting α-Syn mRNA translation. Conversely, IRP1 binds with α-Syn IRE RNA well at low cellular iron levels, blocking ribosome and initiation factor binding to regulate the production of neurotoxic proteins. IRP1’s separation from α-Syn IRE RNA is promoted by iron binding, which also alters the structure of the α-Syn RNA/IRP1 complex. By promoting the overproduction of the neurotoxic α-synuclein, this complex helps Parkinson’s disease progress through protein aggregation. α-Syn IRE RNA also contributes to iron regulation in the brain, as evidenced by the fact that the equilibrium binding of both IRE RNAs (α-Syn IRE RNA versus ferritin IRE RNA) is comparable for IRP1 binding. We have previously demonstrated that iron promotes IRP1 release from the ferritin IRE/IRP1 interaction, enabling ribosome interaction. As a result, ferritin mRNA translation is improved [40,67,75]. Elevated iron levels have been found in the central nervous systems of PD patients [76]. By altering its structure, iron overload decreases the binding affinity of IRE RNA/IRP1. This, in turn, enhances translation initiation factors and/or ribosomal binding affinity, which in turn enhances the translation of α-synuclein mRNA.

## 5. Conclusions

The molecular characteristics of α-Syn IRE RNA-IRP1 binding were revealed by the use of CD and fluorescence spectroscopy in conjunction with RNA structural studies. The molecular complex of α-Syn IRE RNA and IRP1 must be optimized by considering a number of factors, such as fluorescence spectra, binding constants, binding locations, Gibb’s free energy, and structural alterations. α-syn IRE RNA’s binding affinity with IRP1 was demonstrated by the fluorescence analysis (*K*_a_ = 21 × 10^−6^ M^−1^), underscoring the strength of the association. Iron significantly reduces the affinity of α-syn IRE RNA/IRP1. Furthermore, the Van’t Hoff equation provided us with a negative ΔG value, indicating that the reaction was thermodynamically advantageous and spontaneous. Using the thermodynamics of α-Syn IRE RNA with IRP1, data revealed that the primary interactions between RNA and proteins are hydrogen bonding as well as van der Waal’s interactions. When α-Syn IRE RNA was present, IRP1 far-UV CD spectra moved upward, indicating that the IRP1•α-Syn IRE RNA complex was formed. Fluorescence binding and thermostability results show that α-Syn IRE RNA binds to IRP1 with a high binding energy and forms many tight contacts with important residues using different bonding forces.

In conclusion, this study illustrates how α-Syn IRE RNA binds to IRP1. This is the first study of its sort to examine the interaction mechanism between the therapeutically relevant α-Syn mRNA and IRP1 protein, a key player in PD. Knowing the mechanism of interaction between α-Syn IRE RNA and IRP1 will help us to understand forces that cause this connection and the specifics of how iron causes the α-Syn IRE RNA•IRP1 complex to become unstable. This study provides insights into the binding mechanism of α-Syn IRE RNA with IRP1 and can be very helpful for treating Parkinson’s disease by controlling the α-synuclein mRNA translation. Further investigation into the distinct structural characteristics and functional implications of α-Syn IRE RNA with translation initiation factors may clarify their biological relevance and their uses in targeting the iron response element to regulate α-Syn translation.

## Figures and Tables

**Table 1:** Temperature-dependent dissociation constants (*K*_d_) of α-synuclein IRE RNA interaction with IRP1 in the absence and presence of iron.

Complex			*K_d_* (nM)			
	5 °C	10 °C	15 °C	20 °C	25 °C	30 °C
α-Syn IRE∙IRP1	13.5 ± 0.6	21.9 ± 0.8	27.8 ± 1.2	38.9 ± 1.5	47.6 ± 1.7	63.8 ± 3.2
α-Syn IRE∙IRP1-Fe^2+^	29.6 ± 1.5	54.4 ± 2.6	88.7 ± 3.8	123.6 ± 6.3	157 ± 7.7	198.7 ± 7.4

**Table 2:** Fe^2+^ changes the thermodynamic parameters, enthalpy (Δ*H*), entropy (Δ*S*), and Gibb’s free energy (Δ*G*), of the α-synuclein IRE RNA binding to IRP1.

Complex	Δ*H* (kJ/mol)	Δ*S* (J/mol/K)	Δ*G* (kJ/mol)	TΔS/Δ*G* (%)
α-Syn IRE∙IRP1	−42.2 ± 2.3	7.0 ± 0.4	−43.7 ± 2.7	4.8
α-Syn IRE∙IRP1-Fe^2+^	−53.0 ± 4.6	48.0 ± 2.7	−36.3 ± 2.1	39.4

## Data Availability

The corresponding author can provide the datasets used and/or analyzed in this study upon reasonable request.

## References

[1] Tysnes O.B., Storstein A. (2017). Epidemiology of Parkinson’s disease. J. Neural Transm..

[2] Spillantini M.G., Schmidt M.L., Lee V.M.Y., Trojanowski J.Q., Jakes R., Goedert M. (1997). α-synuclein in Lewy bodies. Nature.

[3] Morris H.R., Spillantini M.G., Sue C.M., Williams-Gray C.H. (2024). The pathogenesis of Parkinson’s disease. Lancet.

[4] Spillantini M.G., Goedert M. (2000). The α-synucleinopathies: Parkinson’s disease, dementia with Lewy bodies, and multiple system atrophy. Ann. N. Y. Acad. Sci..

[5] Polymeropoulos M.H., Lavedan C., Leroy E., Ide S.E., Dehejia A., Dutra A., Dutra A., Pike B., Root H., Rubenstein J. (1997). Mutation in the α-synuclein gene identified in families with Parkinson’s disease. Science.

[6] Goedert M., Jakes R., Spillantini M.G. (2017). The Synucleinopathies: Twenty Years On. J. Parkinsons Dis..

[7] Zarranz J.J., Alegre J., Gómez-Esteban J.C., Lezcano E., Ros R., Ampuero I., Vidal L., Hoenicka J., Rodriguez O., Atarés B. (2004). The new mutation, E46K, of α-synuclein causes Parkinson and Lewy body dementia. Ann. Neurol..

[8] Appel-Cresswell S., Vilarino-Guell C., Encarnacion M., Sherman H., Yu I., Shah B., Weir D., Thompson C., Szu-Tu C., Trinh J. (2013). α-synuclein p.H50Q, a novel pathogenic mutation for Parkinson’s disease. Mov. Disord..

[9] Chartier-Harlin M.C., Kachergus J., Roumier C., Mouroux V., Douay X., Lincoln S., Levecque C., Larvor L., Andrieux J., Hulihan M. (2004). Causal relation between α-synuclein gene duplication and familial Parkinson’s disease. Lancet.

[10] Zhang P., Park H.-J., Zhang J., Junn E., Andrews R.J., Velagapudi S.P., Abegg D., Vishnu K., Costales M.G., Childs-Disney J.L. (2020). Translation of the intrinsically disordered protein α-synuclein is inhibited by a small molecule targeting its structured mRNA. Proc. Natl. Acad. Sci. USA.

[11] Velagapudi S.P., Gallo S.M., Disney M.D. (2014). Sequence-based design of bioactive small molecules that target precursor microRNAs. Nat. Chem. Biol..

[12] Luk K.C., Kehm V., Carroll J., Zhang B., O’Brien P., Trojanowski J.Q., Lee V.M.Y. (2012). Pathological α-synuclein transmission initiates Parkinson-like neurodegeneration in nontransgenic mice. Science.

[13] Goldstein D.S., Isonaka R., Lamotte G., Kaufmann H. (2021). Different phenoconversion pathways in pure autonomic failure with versus without Lewy bodies. Clin. Auton. Res..

[14] Nechushtai L., Frenkel D., Pinkas-Kramarski R. (2023). Autophagy in Parkinson’s Disease. Biomolecules.

[15] Malpartida A.B., Williamson M., Narendra D.P., Wade-Martins R., Ryan B.J. (2021). Mitochondrial Dysfunction and Mitophagy in Parkinson’s Disease: From Mechanism to Therapy. Trends Biochem. Sci..

[16] Schmukler E., Pinkas-Kramarski R. (2020). Autophagy induction in the treatment of Alzheimer’s disease. Drug Dev. Res..

[17] Burré J., Sharma M., Südhof T.C. (2018). Cell Biology and Pathophysiology of α-Synuclein. Cold Spring Harb. Perspect. Med..

[18] Bartels T., Choi J.G., Selkoe D.J. (2011). α-Synuclein occurs physiologically as a helically folded tetramer that resists aggregation. Nature.

[19] Fusco G., De Simone A., Gopinath T., Vostrikov V., Vendruscolo M., Dobson C.M., Veglia G. (2014). Direct observation of the three regions in α-synuclein that determine its membrane-bound behaviour. Nat. Commun..

[20] Vamvaca K., Volles M.J., Lansbury P.T. (2009). The first N-terminal amino acids of α-synuclein are essential for α-helical structure formation in vitro and membrane binding in yeast. J. Mol. Biol..

[21] Nielsen M.S., Vorum H., Lindersson E., Jensen P.H. (2001). Ca^2+^ binding to α-synuclein regulates ligand binding and oligomerization. J. Biol. Chem..

[22] Lautenschläger J., Stephens A.D., Fusco G., Stroehl F., Curry N., Zacharopoulou M., Michel C.H., Laine R., Nespovitaya N., Fantham M. (2018). C-terminal calcium binding of α-synuclein modulates synaptic vesicle interaction. Nat. Commun..

[23] Carapeto A.P., Marcuello C., Faísca P.F.N., Rodrigues M.S. (2024). Morphological and Biophysical Study of S100A9 Protein Fibrils by Atomic Force Microscopy Imaging and Nanomechanical Analysis. Biomolecules.

[24] Fleming R.E., Ponka P. (2012). Iron overload in human disease. N. Engl. J. Med..

[25] Khan M.A. (2024). Targeting iron responsive elements (IREs) of APP mRNA into novel therapeutics to control the translation of amyloid-β precursor protein in Alzheimer’s disease. Pharmaceuticals.

[26] Kruszewski M. (2003). Labile iron pool: The main determinant of cellular response to oxidative stress. Mutat. Res..

[27] Uversky V.N., Li J., Fink A.L. (2001). Evidence for a partially folded intermediate in α-synuclein fibril formation. J. Biol. Chem..

[28] Dexter D.T., Carayon A., Javoy-Agid F., Agid Y., Wells F.R., Daniel S.E., Lees A.J., Jenner P., Marsden C.D. (1991). Alterations in the levels of iron, ferritin and other trace metals in Parkinson’s disease and other neurodegenerative diseases affecting the basal ganglia. Brain.

[29] Faucheux B.A., Martin M., Beaumont C., Hunot S., Hauw J., Agid Y., Hirsch E.C. (2002). Lack of up-regulation of ferritin is associated with sustained iron regulatory protein-1 binding activity in the substantia nigra of patients with Parkinson’s disease. J. Neurochem..

[30] Febbraro F., Giorgi M., Caldarola S., Loreni F., Romero-Ramos M. (2012). α-Synuclein expression is modulated at the translational level by iron. NeuroReport.

[31] McDowall J.S., Brown D.R. (2016). α-synuclein: Relating metals to structure, function and inhibition. Met. Integr. Biometal Sci..

[32] Zhou Z.D., Tan E.-K. (2017). Iron regulatory protein (IRP)-iron responsive element (IRE) signaling pathway in human neurodegenerative diseases. Mol. Neurodegener..

[33] Friedlich A.L., Tanzi R.E., Rogers J.T. (2007). The 5′-untranslated region of Parkinson’s disease α-synuclein messengerRNA contains a predicted iron responsive element. Mol. Psychiatry.

[34] Rogers J.T., Randall J.D., Cahill C.M., Eder P.S., Huang X., Gunshin H., Leiter L., McPhee J., Sarang S.S., Utsuki T. (2002). An iron-responsive element type II in the 5′-untranslated region of the Alzheimer’s amyloid precursor protein transcript. J. Biol. Chem..

[35] Selezneva A.I., Cavigiolio G., Theil E.C., Walden W.E., Volz K. (2006). Crystallization and preliminary X-ray diffraction analysis of iron regulatory protein 1 in complex with ferritin IRE RNA. Acta Crystallogr. Sect. F Struct. Biol. Cryst. Commun..

[36] Brazzolotto X., Timmins P., Dupont Y., Moulis J.M. (2002). Structural changes associated with switching activities of human iron regulatory protein 1. J. Biol. Chem..

[37] Khan M.A., Mohammad T., Malik A., Hassan M.I., Domashevskiy A.V. (2023). Iron response elements (IREs)-mRNA of Alzheimer’s amyloid precursor protein binding to iron regulatory protein (IRP1): A combined molecular docking and spectroscopic approach. Sci. Rep..

[38] Volpon L., Osborne M.J., Topisirovic I., Siddiqui N., Borden K.L. (2006). Cap-free structure of eIF4E suggests a basis for conformational regulation by its ligands. EMBO J..

[39] Khan M.A., Malik A., Domashevskiy A.V., San A., Khan J.M. (2020). Interaction of ferritin iron responsive element (IRE) mRNA with translation initiation factor eIF4F. Spectrochim. Acta Part A Mol. Biomol. Spectrscopy.

[40] Khan M.A., Walden W.E., Goss D.J., Theil E.C. (2009). Direct Fe^2+^ sensing by iron-responsive messenger RNA:repressor complexes weakens binding. J. Biol. Chem..

[41] Ke Y., Wu J., Leibold E.A., Walden W.E., Theil E.C. (1998). Loops and bulge/loops in iron-responsive element isoforms influence iron regulatory protein binding. Fine-tuning of mRNA regulation?. J. Biol. Chem..

[42] Bradford M.M. (1976). A rapid and sensitive method for the quantitation of microgram quantities of protein utilizing the principle of protein-dye binding. Anal. Biochem..

[43] Reuter J.S., Mathews D.H. (2010). RNAstructure: Software for RNA secondary structure prediction and analysis. BMC Bioinform..

[44] Zuker M. (2003). Mfold web server for nucleic acid folding and hybridization prediction. Nucleic Acids Res..

[45] Zhang Y., Wang J., Xiao Y. (2022). 3dRNA: 3D Structure Prediction from Linear to Circular RNAs. J. Mol. Biol..

[46] Biesiada M., Pachulska-Wieczorek K., Adamiak R.W., Purzycka K.J. (2016). RNAComposer and RNA 3D structure prediction for nanotechnology. Methods.

[47] Khan M.A., Walden W.E., Theil E.C., Goss D.J. (2017). Thermodynamic and kinetic analyses of iron response element (IRE)-mRNA binding to iron regulatory protein, IRP1. Sci. Rep..

[48] Chen Y.H., Yang J.T., Martinez H.M. (1972). Determination of the secondary structures of proteins by circular dichroism and optical rotatory dispersion. Biochemistry.

[49] Volz K. (2008). The functional duality of iron regulatory protein 1. Curr. Opin. Struct. Biol..

[50] Theil E.C. (2007). Coordinating responses to iron and oxygen stress with DNA and mRNA promoters: The ferritin story. Biometals Int. J. Role Met. Ions Biol. Biochem. Med..

[51] Olivares D., Huang X., Branden L., Greig N.H., Rogers J.T. (2009). Physiological and pathological role of α-synuclein in Parkinson’s disease through iron mediated oxidative stress; the role of a putative iron-responsive element. Int. J. Mol. Sci..

[52] Rogers J.T., Randall J.D., Eder P.S., Huang X., Bush A.I., Tanzi R.E., Venti A., Payton S.M., Giordano T., Nagano S. (2002). Alzheimer’s disease drug discovery targeted to the APP mRNA 5′untranslated region. J. Mol. Neurosci..

[53] Leipuviene R., Theil E.C. (2007). The family of iron responsive RNA structures regulated by changes in cellular iron and oxygen. Cell. Mol. Life Sci..

[54] Muckenthaler M.U., Galy B., Hentze M.W. (2008). Systemic iron homeostasis and the iron-responsive element/iron-regulatory protein (IRE/IRP) regulatory network. Annu. Rev. Nutr..

[55] Shen M., Goforth J.B., Eisenstein R.S. (2023). Iron-dependent post transcriptional control of mitochondrial aconitase expression. Met. Integr. Biometal Sci..

[56] Cho H.-H., Cahill C.M., Vanderburg C.R., Scherzer C.R., Wang B., Huang X., Rogers J.T. (2010). Selective translational control of the Alzheimer amyloid precursor protein transcript by iron regulatory protein-1. J. Biol. Chem..

[57] Mikkilineni S., Cantuti-Castelvetri I., Cahill C.M., Balliedier A., Greig N.H., Rogers J.T. (2012). The anticholinesterase phenserine and its enantiomer posiphen as 5′untranslated-region-directed translation blockers of the Parkinson’s alpha synuclein expression. Park. Dis..

[58] Goforth J.B., Anderson S.A., Nizzi C.P., Eisenstein R.S. (2010). Multiple determinants within iron-responsive elements dictate iron regulatory protein binding and regulatory hierarchy. RNA.

[59] Piccinelli P., Samuelsson T. (2007). Evolution of the iron-responsive element. RNA.

[60] Gao K., Oerlemans R., Groves M.R. (2020). Theory and applications of differential scanning fluorimetry in early-stage drug discovery. Biophys. Rev..

[61] Khrapunov S. (2009). Circular dichroism spectroscopy has intrinsic limitations for protein secondary structure analysis. Anal. Biochem..

[62] Rodger A., Marrington R., Roper D., Windsor S. (2005). Circular dichroism spectroscopy for the study of protein-ligand interactions. Methods Mol. Biol..

[63] Rabbani G., Kaur J., Ahmad E., Khan R.H., Jain S.K. (2014). Structural characteristics of thermostable immunogenic outer membrane protein from Salmonella enterica serovar Typhi. Appl. Microbiol. Biotechnol..

[64] Khan M.A., Kumar Y., Tayyab S. (2002). Bilirubin binding properties of pigeon serum albumin and its comparison with human serum albumin. Int. J. Biol. Macromol..

[65] Cahill C.M., Lahiri D.K., Huang X., Rogers J.T. (2009). Amyloid precursor protein and alpha synuclein translation, implications for iron and inflammation in neurodegenerative diseases. Biochim. Biophys. Acta.

[66] Hintze K.J., Theil E.C. (2005). DNA and mRNA elements with complementary responses to hemin, antioxidant inducers, and iron control ferritin-L expression. Proc. Natl. Acad. Sci. USA.

[67] Ma J., Haldar S., Khan M.A., Das Sharma S., Merrick W.C., Theil E.C., Goss D.J. (2012). Fe^2+^ binds iron responsive element-RNA, selectively changing protein-binding affinities and regulating mRNA repression and activation. Proc. Natl. Acad. Sci. USA.

[68] Ross P.D., Subramanian S. (1981). Thermodynamics of protein association reactions: Forces contributing to stability. Biochemistry.

[69] Khan M.A. (2022). Ferritin iron responsive elements (IREs) mRNA interacts with eIF4G and activates in vitro translation. Front. Biosci. (Elite Ed.).

[70] Tayyab S., Sam S.E., Kabir M.Z., Ridzwan N.F.W., Mohamad S.B. (2019). Molecular interaction study of an anticancer drug, ponatinib with human serum albumin using spectroscopic and molecular docking methods. Spectrochim. Acta Part A Mol. Biomol. Spectrosc..

[71] Kuntz I.D., Chen K., Sharp K.A., Kollman P.A. (1999). The maximal affinity of ligands. Proc. Natl. Acad. Sci. USA.

[72] Williams D.J., Hall K.B. (1996). RNA hairpins with non-nucleotide spacers bind efficiently to the human U1A protein. J. Mol. Biol..

[73] Bannister W.H., Bannister J.V. (1974). Evidence for the validity of three-component fitting of protein circular dichroism spectra. Z. Naturforsch. C Biosci..

[74] Masuda T., Goto F., Yoshihara T., Mikami B. (2010). The universal mechanism for iron translocation to the ferroxidase site in ferritin, which is mediated by the well conserved transit site. Biochem. Biophys. Res. Commun..

[75] Khan M.A., Domashevskiy A.V. (2021). Iron enhances the binding rates and translational efficiency of iron responsive elements (IREs) mRNA with initiation factor eIF4F. PLoS ONE.

[76] Ward R.J., Zucca F.A., Duyn J.H., Crichton R.R., Zecca L. (2014). The role of iron in brain ageing and neurodegenerative disorders. Lancet Neurol..

